# Micro–RNA-126 Reduces the Blood Thrombogenicity in Diabetes Mellitus via Targeting of Tissue Factor

**DOI:** 10.1161/ATVBAHA.115.306094

**Published:** 2016-05-25

**Authors:** Marco Witkowski, Alice Weithauser, Termeh Tabaraie, Daniel Steffens, Nicolle Kränkel, Mario Witkowski, Bernd Stratmann, Diethelm Tschoepe, Ulf Landmesser, Ursula Rauch-Kroehnert

**Affiliations:** From the Charité Centrum 11, Department of Cardiology, Charité–Universitätsmedizin, Berlin, Germany; Research Centre Immunology and Institute of Medical Microbiology and Hygiene, University of Mainz Medical Centre, Mainz, Germany; and Heart and Diabetes Center NRW, Ruhr University of Bochum, Bad Oeynhausen, Germany.

**Keywords:** diabetes mellitus, endothelial cells, monocytes, thromboplastin, untranslated regions

## Abstract

**Objective—:**

Diabetes mellitus involves vascular inflammatory processes and is a main contributor to cardiovascular mortality. Notably, heightened levels of circulating tissue factor (TF) account for the increased thrombogenicity and put those patients at risk for thromboembolic events. Here, we sought to investigate the role of micro-RNA (miR)–driven TF expression and thrombogenicity in diabetes mellitus.

**Approach and Results—:**

Plasma samples of patients with diabetes mellitus were analyzed for TF protein and activity as well as miR-126 expression before and after optimization of the antidiabetic treatment. We found low miR-126 levels to be associated with markedly increased TF protein and TF-mediated thrombogenicity. Reduced miR-126 expression was accompanied by increased vascular inflammation as evident from the levels of vascular adhesion molecule-1 and fibrinogen, as well as leukocyte counts. With optimization of the antidiabetic treatment miR-126 levels increased and thrombogenicity was reduced. Using a luciferase reporter system, we demonstrated miR-126 to directly bind to the *F3*-3′-untranslated region, thereby reducing TF expression both on mRNA and on protein levels in human microvascular endothelial cells as well as TF mRNA and activity in monocytes.

**Conclusions—:**

Circulating miR-126 exhibits antithrombotic properties via regulating post-transcriptional TF expression, thereby impacting the hemostatic balance of the vasculature in diabetes mellitus.

## Abstract 2

Supplemental Digital Content is available in the text.

Being a metabolic disorder with increasing incidence, diabetes mellitus is a main contributor to cardiovascular mortality mostly caused by thromboembolic complications.^1^ Inflammatory processes within the vasculature and blood are involved in the pathogenesis of the disease promoting endothelial dysfunction and its atherothrombotic complications.^2,3^

Tissue factor (TF), the primary initiator of blood coagulation, is released from the vasculature and circulating cells on inflammatory stimuli.^4,5^ Increased TF levels in the blood are known to be associated with cardiovascular mortality.^6^ Notably, the membrane-bound full-length (fl) TF accounts for its procoagulant properties. In contrast, the alternatively spliced (as) TF is much less procoagulant and promotes angiogenesis and cell survival/proliferation. Particularly in the patients with diabetes mellitus, circulating TF is upregulated and contributes to a procoagulant state.^7,8^ On glycemic control, circulating TF expression has been shown to decrease, leading to a reduction in the thrombogenicity.^9^

Recently, micro-RNA (miRs) have emerged as novel factors involved in vascular inflammation and TF biology. For instance, miR-19a and miR-19b bind to the TF transcript and were shown to control the TF expression in human cancer cells and endothelial cells.^10,11^ MiRs were also implicated in the development of diabetes mellitus and diabetic late complications.^12–14^ Importantly, the endothelial-specific miR-126 was found to be downregulated in individuals with diabetes mellitus and also in those susceptible to diabetes mellitus.^12,15^ Moreover, miR-126 levels negatively correlate with the atherogenicity of patient-derived triglyceride-rich lipoproteins.^16^ Within the vessel, miR-126 is involved in vascular repair^17^ and has anti-inflammatory properties caused by the inhibition of endothelial vascular adhesion molecule (VCAM)-1 expression.^18^ Experimental studies have shown that miR-126 also plays a role in prevention from complications associated with diabetes mellitus, such as pathological angiogenesis. Furthermore, miR-126 reduces diabetic retinopathy in a mouse model through the inhibition of vascular endothelial growth factor.^19^ Whether altered miR-126 expression contributes to the hypercoagulability in patients with diabetes mellitus is to date unknown.

## Materials and Methods

Materials and Methods are available in the online-only Data Supplement.

## Results

### MiR-126 Correlates With TF and Thrombogenicity in Patients With Diabetes Mellitus

**Figure 1.**
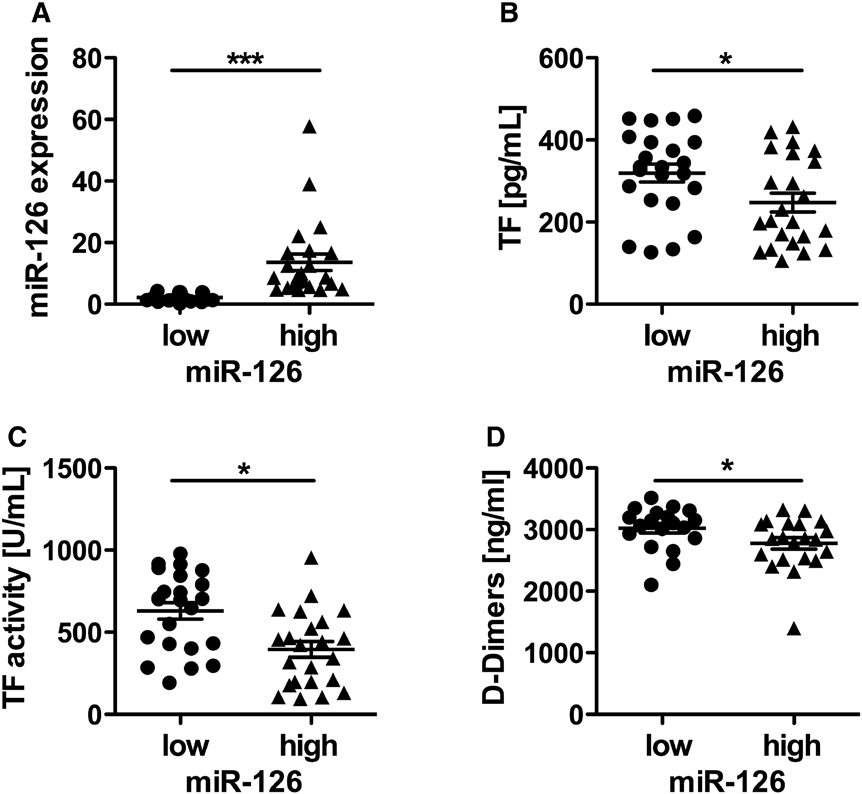
High micro-RNA (miR)-126 expression is associated with reduced tissue factor (TF) protein and TF-mediated thrombogenicity in patients with diabetes mellitus. **A**, Expression of miR-126 in both groups with either low or high miR-126 expression. Differences in (**B**) TF protein and (**C**) TF activity in plasma depending on low or high miR-126 plasma expression. **D**, Levels of D-dimers in the patients of both groups. Data are expressed as mean±SEM. n= 46, for D-dimers n=40, *P<0.05, ***P<0.0001.

In this study, 46 patients (34 men and 12 women) with known diabetes mellitus and poor glycemic control (mean glycated hemoglobin [HbA1c] of 8.3%) were examined. According to the plasma miR-126 expression, the patients were divided into 2 groups with either high miR-126 expression above the median or low miR-126 expression below the median (Table 1). There were no differences in the 2 groups on diabetes mellitus duration, fasting blood glucose, or HbA1c. However, in the group with high miR-126 expression, a higher percentage of treatment with metformin or sulfonylurea was seen. Plasma specimens were obtained before and after intensified antidiabetic treatment. To assess the impact of miR-126 on TF biology, TF protein amounts were analyzed using a TF ELISA from American Diagnostica for total TF expression and an flTF-specific ELISA from Hyphen Biomed. A factor Xa chromogenic assay was used to quantify the TF activity. miR-126 levels were significantly different in both groups (Figure 1A). The patients with a high miR-126 expression showed a significantly lower protein level of TF in blood than the patients with a low miR-126 expression (Figure 1B). This association was confirmed using the flTF ELISA (data not shown). In line, TF activity was significantly decreased in patients with high miR-126 levels when compared with the group with low miR-126 levels (Figure 1C). Moreover, we observed significantly lower levels of D-dimers in the patients with high miR-126 expression than in the patients with low miR-126 expression (Figure 1D).

**Table 1.**
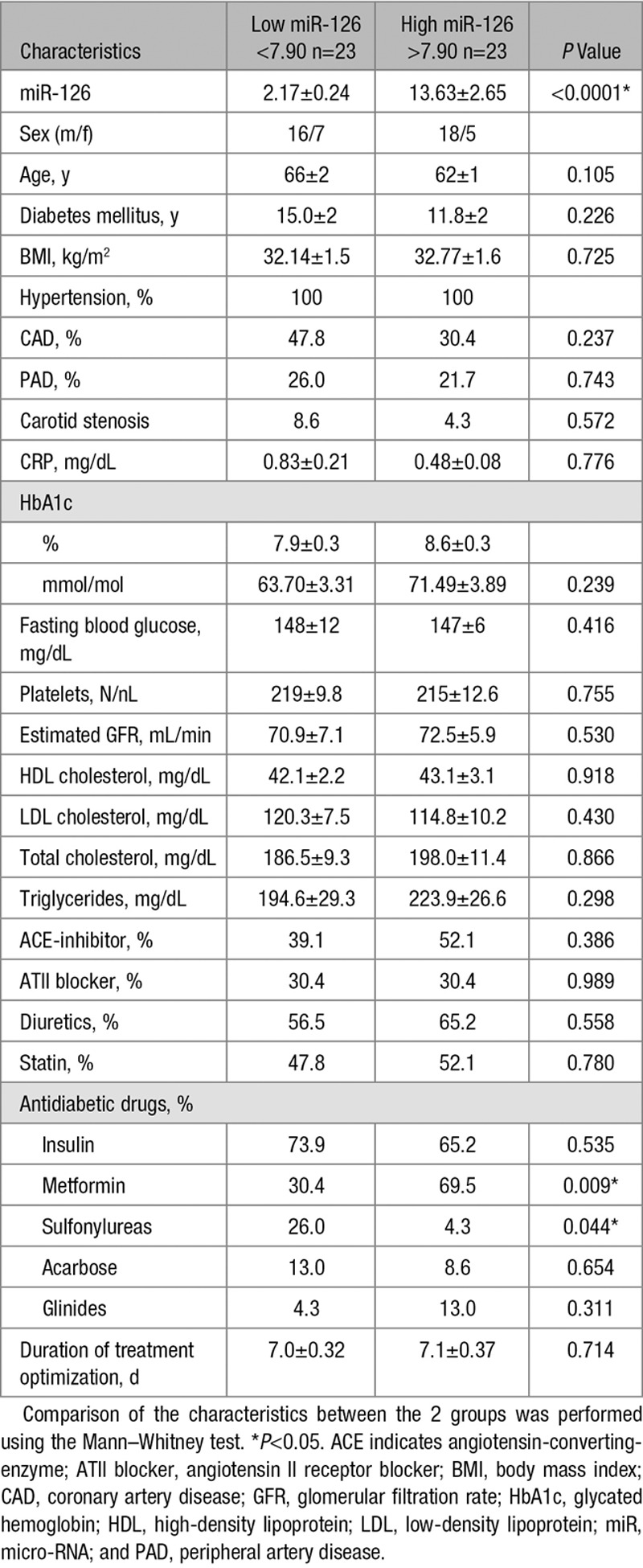
Patient Characteristics in Both Groups

### Endothelial miR-126 and Vascular Inflammation

**Figure 2.**
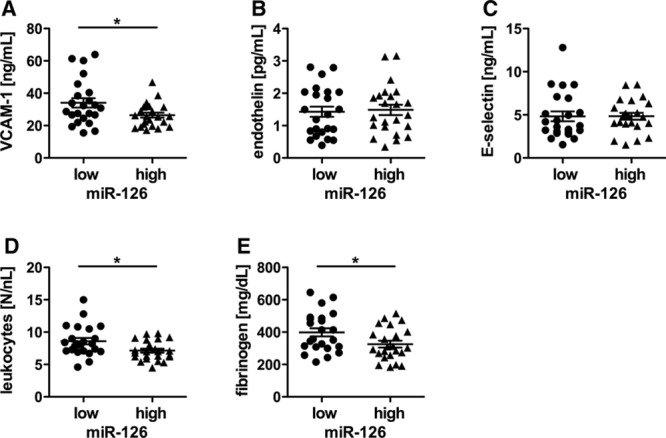
Micro-RNA (miR)-126 is associated with reduced vascular inflammation in patients with diabetes mellitus. **A**, Expression levels of the inflammatory proteins vascular adhesion molecule (VCAM)-1, (**B**) endothelin, (**C**) E-selectin, (**D**) leukocyte count as well as (**E**) fibrinogen in patients with either low or high miR-126 expression. Data are expressed as mean±SEM. n= 46, *P<0.05.

To further investigate the role of miR-126 in the pathogenesis of diabetes mellitus, markers of vascular inflammation were assessed. In the group of patients with high miR-126 expression, we observed a significant lower leukocyte count than in the group with low miR-126 expression (Figure 2D). In addition, the levels of fibrinogen were significantly reduced in the group with high miR-126 levels compared with the group with low miR-126 levels (Figure 2E). Moreover, VCAM-1 but not intercellular adhesion molecule-1 (data not shown), endothelin, or E-selectin were significantly diminished in the group with high miR-126 compared with low miR-126 expression (Figure 2A–2C), reflecting less endothelial dysfunction.

### Optimized Antidiabetic Treatment Is Associated With an Upregulation of miR-126 and Reduction in Thrombogenicity

**Figure 3.**
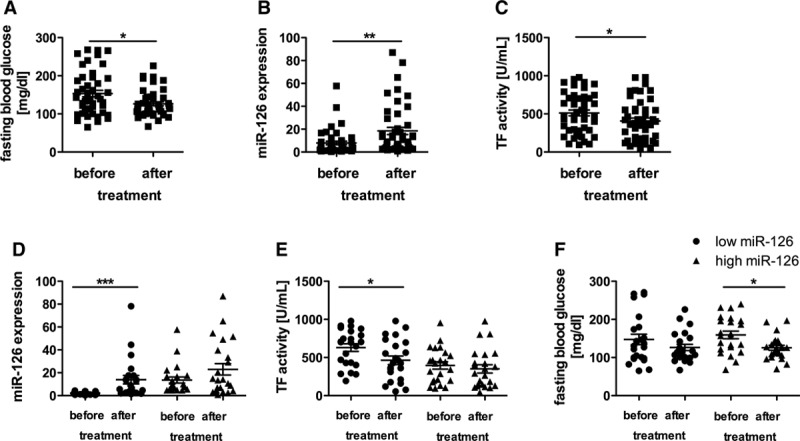
The optimized antidiabetic treatment is associated with upregulation of micro-RNA (miR)-126 levels and a reduction in tissue factor (TF)–mediated thrombogenicity**. A**, Fasting blood glucose levels, (**B**) miR-126 expression, and (**C**) TF activity in all patients before and after optimizing of anti-diabetic treatment. Changes in miR-126 expression (**D**), TF activity (**E**), and fasting blood glucose levels (**F**) on optimized antidiabetic treatment in both, patients with low and high initial miR-126 expression on admission. Data are expressed as mean±SEM. n= 46, *P<0.05, **P<0.01, ***P<0.0001.

During the course of the study, the patients received an optimization of their antidiabetic treatment based on dose adaptation of their medication to obtain a better glucose control (Table 2). The glycemic control was improved after this treatment period (Figure 3A). Moreover, the optimized treatment resulted in an increase in miR-126 expression when compared with time of hospital admission (Figure 3B). The TF activity was diminished by improved therapy (Figure 3C).

On optimized treatment, the expression of miR-126 was upregulated only in those patients with initial low miR-126 expression (Figure 3D). This increase in miR-126 expression was associated with a marked reduction in TF activity only in patients with initial low miR-126 expression (Figure 3E). In contrast, patients with already high miR-126 expression on admission exhibited initially lower TF activity that was not further reduced on treatment optimization (Figure 3E). Moreover, the patients with high miR-126 expression had lower fasting glucose levels on improved treatment than the patients with low-miR-126 (Figure 2F).

### miR-126 Downregulates Alternatively Spliced TF and flTF in Human Microvascular Endothelial Cells-1

**Figure 4.**
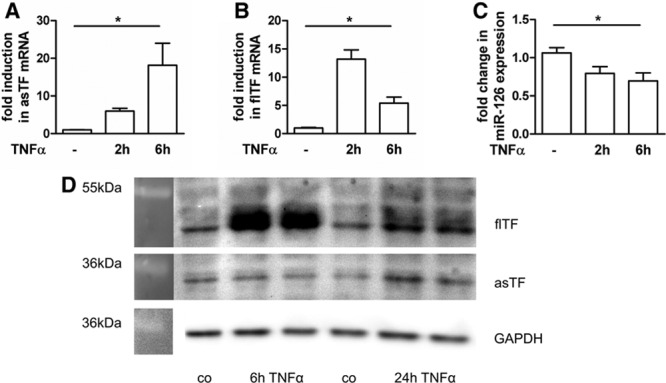
Increased full-length tissue factor (flTF) and alternatively spliced (as) TF expression in endothelial cells after stimulation with tumor necrosis factor (TNF)-α. Human microvascular endothelial cells were cultured for 24 h and then treated with 10 ng/mL TNFα. Gene expression was determined for (**A**) alternatively spliced TF, (**B**) flTF, and (**C**) miR-126 2 and 6 h after stimulation with TNFα. GAPDH was used as a house-keeping gene. Data are represented as mean±SEM. *P<0.05, n≥3. **D**, A representative Western blot shows protein expression 6 and 24 h post TNFα stimulation. GAPDH was used as loading control (n=3, performed in duplicates).

In human microvascular endothelial cells (HMEC)-1, the mRNA and protein expression of both, asTF and flTF, were significantly upregulated on stimulation with tumor necrosis factor (TNF)-α (Figure 4A, 4B, and 4D). TNFα also increased the release of microvesicles in HMEC-1 (Figure IIIA in the online-only Data Supplement). The miR-126 expression was reduced by TNFα treatment for 2 and 6 hours (Figure 4C). In the following experiments, TNFα served as an inducer for TF expression reflecting a model of endothelial inflammation.

**Figure 5.**
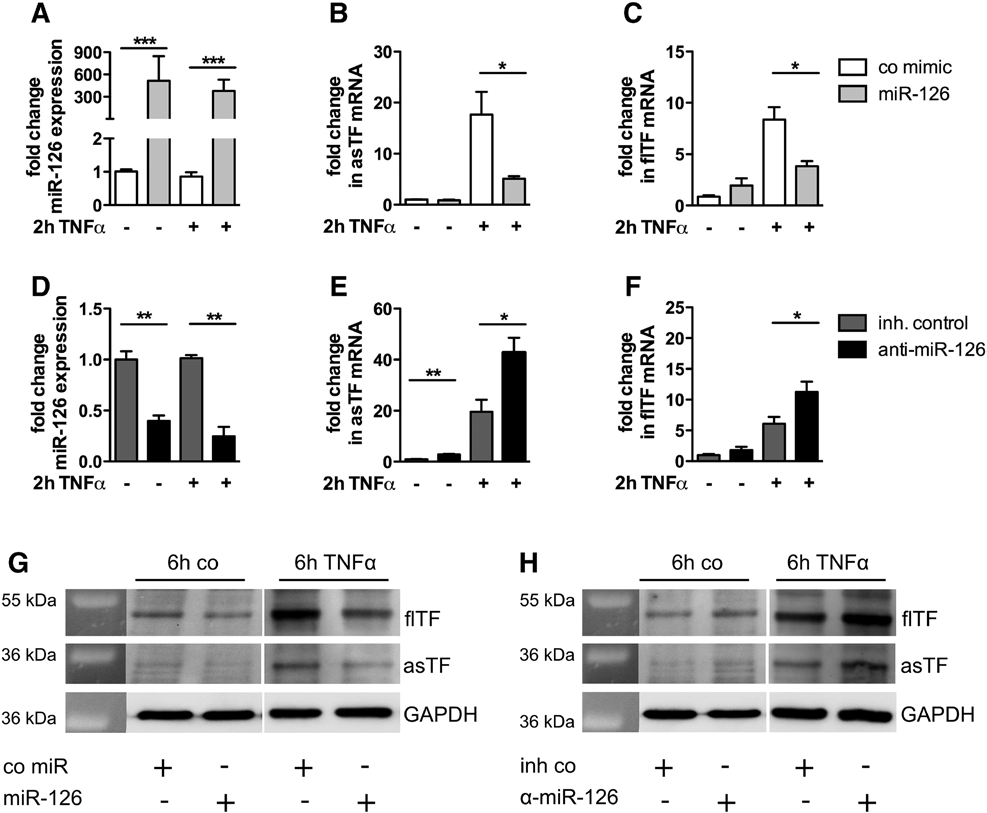
Micro-RNA (miR)-126 reduces the expression of both alternatively spliced tissue factor (asTF) and full-length (fl) TF in human microvascular endothelial cells (HMEC) after stimulation with tumor necrosis factor (TNF)-α. HMECs were cultured for 24 h and then transfected with a control mimic or a miR-126 mimic (**A**–**C**) as well as an inhibitor control or anti–miR-126 (**D**–**F**). The transfection efficiency was confirmed by real-time TaqMan polymerase chain reaction (**A** and **D**). Twenty-four hours post transfection, the cells were stimulated with 10 ng/mL TNFα. Gene expression was determined for asTF (**B** and **E**) and flTF (**C** and **F**) 2 h after stimulation with TNFα. GAPDH was used as house-keeping gene. Data are represented as mean±SEM. *P<0.05, **P<0.01, ***P<0.0001, n≥3. Representative western blots show the protein expression of flTF and asTF after transfection with miR-126 (**G**) or anti–miR-126 (**H**) 6 h post TNFα stimulation. GAPDH was used as loading control (n=3).

To assess the impact of miR-126 on the expression of both TF isoforms, HMECs were transfected with miR-126, anti–miR-126 or the respective controls and subsequently stimulated with TNFα. The efficiency of overexpression and silencing of miR-126 was confirmed via real-time polymerase chain reaction (Figure 5A and 5D). Transfection of HMECs with miR-126 or anti–miR-126 also altered the miR-126 expression in released microvesicles (Figure IIIB in the online-only Data Supplement). The expression of asTF was significantly reduced in HMEC-1 cells transfected with miR-126 compared with cells transfected with the control mimic 2 hours post stimulation with TNFα (Figure 5B). Likewise, the expression of flTF was reduced in the presence of a miR-126 mimic when compared with the control mimic (Figure 5C). In contrast, transfection of an anti–miR-126 led to an increase of asTF and flTF expression 2 hours post stimulation with TNFα (Figure 5E and 5F). Because unstimulated cells expressed almost no TF, we were not able to observe a relevant alteration in TF isoform expression on transfection of miR-126 or anti–miR-126 (Figure 5B, 5C, 5E, and 5F). Western blot analyses showed that transfection of miR-126 led to a significant reduction of flTF on the protein level after 6 hours of TNFα stimulation (Figure 5G). In contrast, transfection of anti-miR-126 exhibited an increase in flTF protein (Figure 5H). After 6 hours of TNFα stimulation, asTF protein was expressed at a lower level. However, a decrease could be observed on transfection with miR-126 (Figure 5G), whereas anti–miR-126 led to an increase (Figure 5H).

### miR-126 Controls TF RNA Expression and Activity in THP-1 Cells

**Figure 6.**
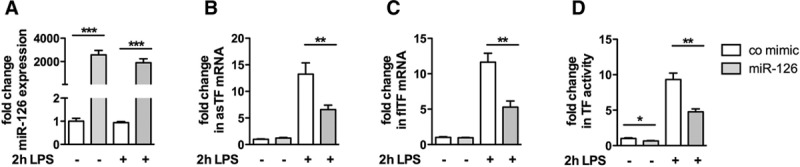
Micro-RNA (miR)-126 decreases tissue factor (TF) transcription and TF-depending FXa generation in THP-1 cells. THP-1 cells were grown over night and then transfected with either a control mimic or a miR-126 mimic for 24 h. The cells were left untreated or stimulated with lipopolysaccharide (LPS; 10 μg/mL) for 2 or 6 h. Subsequently, miR-126 transfection efficiency (**A**), alternatively spliced (as) TF mRNA levels (**B**), full-length (fl) TF mRNA levels (**C**) or the TF activity (**D**) were assessed using real-time TaqMan polymerase chain reaction or a FX chromogenic assay, respectively. Data are represented as mean±SEM. **P<0.01, ***P<0.001, n≥6.

To analyze miR-126–dependent TF expression in monocytic cells, THP-1 cells were used as a model. Because of the low endogenous expression of miR-126 in monocytes, the experiments were performed with a control mimic or a miR-126 mimic. After transfection, the cells were left untreated or stimulated with lipopolysaccharide to induce TF expression. Sufficient transfection efficiency was confirmed by real-time TaqMan polymerase chain reaction (Figure 6A). In quiescent cells, transfection of miR-126 did not cause a significant change in asTF and flTF mRNA levels. However, post stimulation with lipopolysaccharide for 2-hour transfection of miR-126 led to a decrease in asTF and flTF mRNA expression compared with a control mimic (Figure 6B and 6C). To assess the TF-depending thrombogenicity, TF activity was measured in the same cells. On transfection with miR-126, we observed a reduction in TF activity when compared with a control mimic in unstimulated cells and after stimulation with lipopolysaccharide for 6 hours (Figure 6D).

### miR-126 Binds to the 3′ Untranslated Region of the *F3* Transcript

**Figure 7.**
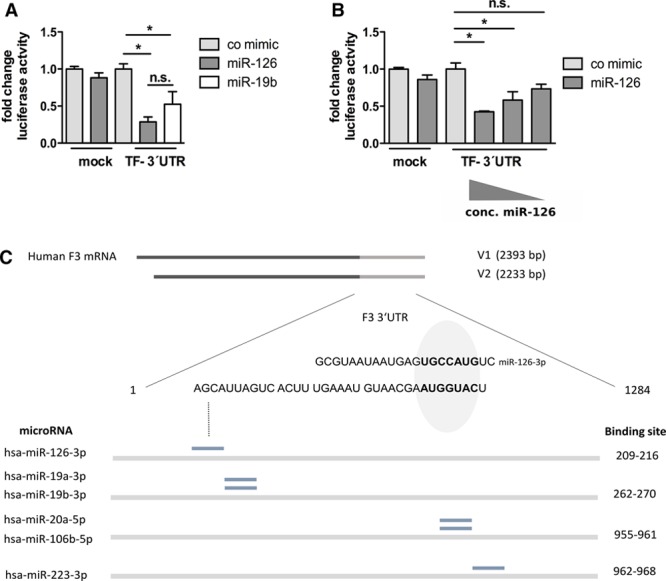
Micro-RNA (miR)-126 targets the 3′-untranslated region (UTR) of the *F3* transcript. **A**, Human embryonic kidney 293 (HEK) cells were cultured for 24 h and then cotransfected with a mock plasmid and 200 μmol/L control mimic or 200 μmol/L miR-126 mimic as well as with a tissue factor (TF)-3′-UTR harboring plasmid and 200 μmol/L control mimic, 200 μmol/L miR-126 mimic or 200 μmol/L miR-19b mimic. Twenty-four hours post transfection, the luciferase activity was measured. **B**, HEK cells were transfected with the TF-3′-UTR plasmid and decreasing amounts of a miR-126 mimic (200, 20, or 2 μmol/L). Subsequently, the luciferase activity was measured. *P<0.05, n=3. **C**, Predicted heteroduplex of miR-126 and the 3′-UTR of the *F3* (TF) transcript in relation to the known proposed miR-binding sites in the human TF transcript. n.s. indicates not significant.

As shown above, miR-126 downregulates the expression of both TF isoforms in human ECs and monocytes. To analyze whether the 3′-untranslated region (UTR) of the TF gene (*F3*) transcript contains binding site(s) for miR-126, in silico analysis was performed (RNA hybrid; http://bibiserv.techfak.uni-bielefeld.de/rnahybrid). We found that the 3′-UTR of the human TF mRNA harbors a binding site for miR-126 (Figure 7C). To confirm a direct binding of miR-126 to the *F3*-3′-UTR, a luciferase assay was performed. Transfection of a miR-126 but not of a control mimic led to a significant decrease in luciferase activity for a luciferase-reporter construct containing the *F3*-3′-UTR (Figure 7A). The decrease in luciferase activity tended to be more pronounced than that exhibited by miR-19b. Accordingly, decreasing concentrations of miR-126 restored the luciferase activity in a concentration-dependent manner (Figure 7B). This indicates that miR-126 directly binds to the 3′-UTR of the *F3* transcript.

## Discussion

In this study, we show that miR-126 correlates with TF protein expression and activity in patients having diabetes mellitus. In ECs and monocytic cells, miR-126 was shown to directly target the *F3*-3′-UTR. Thereby, miR-126 inhibited the TF expression on the post-transcriptional level and seems to contribute to TF-dependent thrombogenicity in diabetes mellitus. Moreover, reduced miR-126 levels were associated with vascular inflammation in patients with diabetes mellitus. The optimization of the antidiabetic treatment was associated with the upregulation of miR-126 plasma levels, which correlated with reduced thrombogenicity in those patients.

**Table 2.**
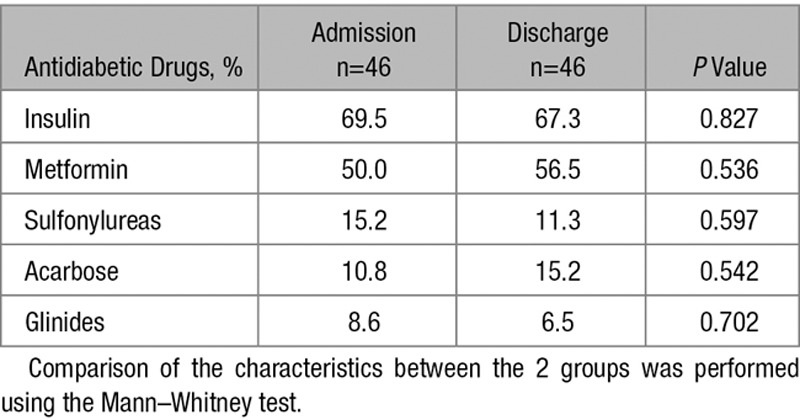
Antidiabetic Treatment on Admission and at Discharge

Circulating TF is increased in diabetes mellitus, especially when cardiovascular disease is present. TF is known to contribute to the elevated thromboembolic risk of those patients.^20–22^ Inflammatory signals within the endothelium account for an increased expression of TF,^4,23^ which is also modulated on the post-transcriptional level.^5,24,25^ Likewise, the endothelial-enriched miR-126 was shown to correlate with diabetes mellitus and coronary artery disease.^12,26^ Up to now, the role of miRs in the pathogenesis of increased thrombogenicity in diabetes mellitus remains unclear. Here, we show that miR-126 reduced the TF expression via binding to its mRNA’s 3′-UTR in human ECs and monocytes. Accordingly, miR-126 levels correlated with TF protein expression and TF-mediated thrombogenicity in a cohort of patients with diabetes mellitus.

In the past, the measurement of TF levels and activity in plasma was challenged by some assay-specific limitations. Bogdanov et al^27^ reported that the measurement of TF activity by the Actichrome kit from American Diagnostica has some limitations because of FVII-independent FXa generation. We therefore used a TF activity assay established and practiced in our laboratory. Moreover, Parhami-Seren et al^28^ suggested that the TF ELISA from American Diagnostica might overestimate the real amount of circulating TF in plasma under certain conditions because in-house TF ELISAs revealed lower amounts for some individuals. We therefore confirmed our original protein data using the Hyphen’s flTF ELISA, which exclusively measured procoagulant flTF in our plasma samples. When applied for isolated human MVs, the Hyphen TF ELISA failed to detect an lipopolysaccharide-induced increase in MV-associated TF.^29^ The authors suggested a possible transfer of lipopolysaccharide-induced MVs to platelets that were then cleared by the preanalytical preparation. However, in this study, we used untreated citrated plasma.

Experimental evidence suggests that miR-126 is released from the vessel wall after damage and may be transferred to ECs, where it exhibits vascular repair through effects, such as modulated expression of sprouty-related protein 1 or stromal cell–derived factor-1.^17,30^ Zampetaki et al^12^ showed that diabetic conditions reduced the levels of miR-126 in plasma and abrogated the vascular protective capacity.^17^ Our results demonstrate that miR-126 also exhibits antithrombotic properties, contributing to the vascular hemostatic balance. A loss of miR-126 caused by hyperglycemia puts patients with diabetes mellitus at a higher risk for thromboembolic complications. In line, downregulated expression of miR-126 in various malignancies may explain, at least in part, the higher risk for thrombosis in patients with cancer.^31–33^

Interestingly, more patients in the group with high miR-126 expression were treated with metformin compared with the group with low miR-126 expression. Metformin is known to reduce the diabetes mellitus–related cardiovascular mortality.^34^ In line, we showed an association of metformin therapy with higher miR-126 expression and reduced thrombogenicity. Nevertheless, in a clinical study, Ortega et al^13^ did not find a significant change in plasma miR-126 expression on treatment with metformin. The small sample size of the work may explain the negative results with unchanged miR-126 expression. Vice versa, more patients in the group with low miR-126 expression were treated with sulfonylurea. Addition of sulfonylurea to the metformin therapy was shown to abrogate the protective effect.^35^ Accordingly, in our patient cohort, sulfonylurea treatment was related to decreased miR-126 expression and increased thrombogenicity. Our findings highlight the differential miR-126 expression on treatment with metformin or sulfonylurea. This may explain the different spectrum of side effects exhibited by those drugs. However, the differences in medication could potentially be confounders as well. Whether specifically the alteration in miR-126 expression in those patients with diabetes mellitus contributes to a beneficial or adverse clinical effect on therapy with metformin or sulfonylurea, respectively, remains to be elucidated. Therefore, larger studies including more patients are warranted.

Our data showed that an optimized antidiabetic treatment was associated with an upregulation of the miR-126 levels in the blood. This was related to a reduction in thrombogenicity in patients with initially low miR-126 expression. The patients with high miR-126 expression exhibited lower fasting glucose levels on improved treatment. This suggests an increase in endothelial miR-126 expression on normalization of blood glucose levels.^17^ Harris et al^36^ demonstrated that miR-126 expression is regulated by the transcription factors ets-1 and ets-2. Interestingly, high-glucose treatment of endothelial cells decreased ets-1 expression.^37^ Hence, the alteration in miR-126 expression could explain that improved glycemic control in diabetes mellitus leads to reduced thrombogenicity.^9^ Vice versa, glycation products were reported to increase TF expression.^9,38^ Our results show an important relationship between upregulation of miR-126 levels and reduction in TF-associated thrombogenicity.

Recently, markers for subclinical inflammation, such as VCAM-1^39^ or the white blood cells,^40^ became relevant to predict a state of prediabetes mellitus before manifestation of the clinical phenotype. Reduced levels of miR-126 were shown to predict the incidence of diabetes mellitus, and miR-126 was proposed as a potential biomarker for diabetes mellitus.^12,41^ In a cohort of patients with diabetes mellitus, we found that in the group with low plasmatic miR-126 expression, leukocyte count and fibrinogen levels were significantly elevated compared with the group with high miR-126 expression. Accordingly, we found the proinflammatory cytokine TNFα to hamper miR-126 expression in ECs. Moreover, VCAM-1 but not endothelin or E-selectin expression was significantly reduced in the group with higher miR-126 expression. This is in line with reports showing that miR-126 directly targets the VCAM-1-transcript in ECs,^18^ modulating its expression in response to triglyceride-rich lipoprotein atherogenicity.^16^ In addition, Srinivasan et al^42^ reported both TF isoforms to ligate β1 integrins on endothelial cells thereby inducing expression of cell adhesion molecules including VCAM-1 and, subsequently, increasing adhesion and transmigration of peripheral blood mononuclear cells. Reduced expression of TF by miR-126 could hence potentially disrupt this pathway and provide an additional mechanism to hamper vascular inflammation.

Our data strengthen the proposed role of miR-126 as a prognostic marker for the development and complications of diabetes mellitus. On the improvement of the glucose metabolism by antidiabetic therapy, miR-126 levels are readily upregulated before markers of vascular inflammation start to change.

## Acknowledgments

We thank Kerstin Kamprath for their technical support in the experiments. Drs Marco Witkowski and Weithauser participated in the design of the study, performed the assays, performed the statistical analysis, and drafted the manuscript both equally. T. Tabaraie performed experiments. Drs Kränkel and Mario Witkowski performed and interpreted the fluorescence-activated cell sorter experiments. Dr Rauch-Kroehnert contributed by designing the study and interpreting the results. Drs Landmesser, Steffens, Stratmann, and Tschoepe critically reviewed and edited the article. All authors read and approved the final article.

## Sources of Funding

This work was supported by a research grant of the Deutsche Forschungsgesellschaft (RA 799/S1).

## Disclosures

None.

## Supplementary Material

## Highlights

- miR-126 directly targets the tissue factor transcript in human endothelial cells and monocytes thereby altering blood thrombogenicity.
- Loss of miR-126 in patients with diabetes mellitus may contribute to their elevated thromboembolic risk.
- Optimized antidiabetic treatment restores miR-126 expression thus reducing thrombogenicity and vascular inflammation.
- Plasma miR-126 may be a prognostic marker for complications of diabetes mellitus.
