# Supplementary material for: Micro–RNA-126 Reduces the Blood Thrombogenicity in Diabetes Mellitus via Targeting of Tissue Factor
Source: Arterioscler Thromb Vasc Biol. 2016 May 25;36(6):1263–71. doi: 10.1161/ATVBAHA.115.306094 (PMC4894779; doi:10.1161/ATVBAHA.115.306094)
Supplement: Supplementary file 1 [file atv-36-1263-s001.pdf]

Material and Methods

Patient study

The study protocol was approved by the local ethics committee and was performed in accordance to the ethics principles in the Declaration of Helsinki. Before participation in the study each patient gave a written informed consent. 46 patients with known diabetes mellitus type 2 (hereinafter referred as to diabetes) admitted at the Diabetes Center NRW Bad Oeynhausen, Germany, were included in the study (1). The reason for admission was insufficient glycemic control. The patients received an optimization of their anti-diabetic treatment based on dose adaptation of their medication in order to obtain a better glucose control. The hospital stay mean time was 1 week (Table 2). Peripheral blood was obtained by venepuncture into heparinized, citrate or EDTA tubes upon admission and discharge. All TF and miR measurements we performed using citrated plasma. miR-126 expression was measured using TaqMan PCR in blood plasma. Protein levels of TF, VCAM-1, intercellular adhesion molecule-1, endothelin, E-selectin, and D-Dimers in plasma were assessed using a specific ELISA system. TF activity in plasma was analyzed by a factor Xa chromogenic assay. To evaluate the influence of miR-126 expression, the patients were divided into two groups according to their miR-126 expression. We defined a group with low miR-126 expression (lower than the median of miR-126 expression of all patients, n=23) and high miR-126 expression (higher than the median of miR-126 expression of all patients, n=23). Table 1 describes the patient characteristics.

ELISA experiments

The TF plasma concentrations were measured by an ELISA from American Diagnostica for total TF and Hyphen Biomed's ELISA for flTF; levels of VCAM-1, Intercellular adhesion molecule-1, endothelin, E-selectin, and D-Dimers were assessed by using a specific ELISA system from American Diagnostica according to the manufacturer's instructions (2).

TF activity

The measurement of bona fide TF activity was performed as described before (3-6). To determine the TF activity, 20µl of citrated plasma was added to 160µl of a solution containing 2nM FVIIa, 150nM FX, and 5mmol/L $CaCl_2$. The generation of FXa was stopped after 30 min by adding EDTA buffer (50mmol/L Bicine, pH 8.5, 100mmol/L NaCl, 25mmol/L EDTA, 1mg/mL BSA). Then spectrozyme (0.5mmol/L final concentration, Sekisui Diagnostics), the chromogenic substrate of FXa, was added to each sample. The optical density was determined at 405nm by using an ELISA plate reader at 37°C (Molecular Devices). TF activity units were assessed by a standard curve. The standard curve is constructed by plotting the mean slope absorbance value measured for each lipidated TF standard against its corresponding concentration [pg/mL]. The activity (generation of FXa) exhibited by 1 pg of lipidated TF corresponds to 1 arbitrary TF-activity unit. The recombinant FVIIa (NovoSeven) was kindly provided by Novo Nordisk.

Cell Culture

Human microvascular endothelial cells (HMEC) from ATCC were maintained in MCBD 131 medium (Gibco) + 10% FBS (Gibco) + 100 U/mL penicillin/streptomycin (PAA) + 2mM L-Glutamin (PAA) + 0,05mg/ml Hydrocortison. HMECs were used for experiments until the 15th passage. Human embryonic kidney (HEK) cells were cultured in DMEM + 10% FBS + 100U/mL penicillin/streptomycin. THP-1 cells were grown in Gibco RPMI 1640 medium (life Technologies) + 10% FBS + 1% penicillin/streptomycin.

Transfection and stimulation experiments

HMEC cells were transfected with 200nM negative control mimic (miRIDIAN micro RNA, Dharmacon), an inhibitor negative control (miRIDIAN micro RNA, Dharmacon), 200nM miR-126 mimic (has-miR-126-3p, MISSION miRNA mimic, Sigma) or 200nM anti-miR-126 (has-miR-126-3p inhibitor, MISSION, Sigma) using the siRNA transfection reagent interferin (VWR) according to manufacturer´s protocol. 24h post transfection, cells were starved in MCBD 131 medium (Gibco) for 1h and then stimulated with 10ng/ml TNFα for 2h for gene expression analysis and 6h or 24h for protein expression of the TF splice variants, respectively. THP-1 cells were stimulated with LPS (10µg/ml) for 2h for mRNA expression analysis and 6h for measurement of TF activity.

Dual luciferase reporter assay

To perform the dual luciferase reporter assay, HEK cells were co-transfected with 200nM control mimic, miR-19b (miR-19b-3p, MISSION miRNA mimic, Sigma) or miR-126 mimic (200nM, 20nM, or 2nM) and a luciferase reporter vector, miTargetTM 3´UTR target clone pEZX-MT01 negative control (GeneCopoeia) or F3-3´UTR (GeneCopoeia) using interferin (VWR). 24h post transfection the luciferase assay was performed using the dual luciferase reporter system (Promega) according to the manufacturer´s protocol.

Real-time PCR and western blot analysis

For real-time PCR, total mRNA was isolated with peqGOLD Trifast (Peqlab) for cell culture experiments or using the mirVana Paris Kit (Life Technologies) for patient blood plasma. Gene expression was determined using our custom FAM-tagged TaqMan® gene expression assays (life Technologies) for flTF and asTF (for details see (7)). The expression of miR126 was analyzed with the FAM tagged TaqMan® gene expression assay (hsa-miR-126-3p – 002228). Relative gene expression was determined via the comparative C(t) (ΔΔCt) method with Glyceraldehyde 3-phosphate dehydrogenase (GAPDH)- Hs99999905_m1 as endogenous control for mRNA and U6 snRNA - 001973 for plasma and cellular miR as endogenous control. Western blots were performed as described before (8). Specific antibodies were used for flTF (#4501, American Diagnostica), asTF (Pineda, custom made, raised in goat), and GAPDH (Calbiochem).

Flow cytometry analysis of microvesicles

Cell supernatants were centrifuged for 10 minutes at 1000g to remove cells. The supernatant was centrifuged again at 20000g for 20 minutes and the resulting pellet washed in PBS. Supernatant or pellets were resuspended in 200µL annexin-binding buffer, including annexinV (Pacific Blue-labelled) as well as antibodies against human CD144 (PE, BioLegend) or VEGF R2 (Fluorescein, R&D Systems). To preclude unspecific signals of antibody-complexes, the staining solution had been cleared by the same centrifugation prior to use. The samples were analysed on an Attune NxT flow cytometer (Life Technologies) using Attune NxT analysis software (Life Technologies). Beads of defined sizes (Megamix, Biocytex, France) were used to determine the cut off at 1µm. The gating procedures are outlined in the supplementary figures I and II.

Statistical analysis

The statistical analyses have been performed using the commercially available software SPSS 22, and/or GraphPad Prism 5. A Mann-Whitney U test has been performed for pairwise comparisons between two independent groups. For comparisons of 1 parameter between more than two groups a 1-way ANOVA was used. Data are represented as mean ± SEM. P-values <0.05 were considered statistically significant.

References:

1. Bobbert P, Rauch U, Stratmann B, Goldin-Lang P, Antoniak S, Bobbert T, Schultheiss HP, Tschoepe D. High molecular weight adiponectin correlates positively with myeloperoxidase in patients with type 2 diabetes mellitus. Diabetes research and clinical practice. 2008;82:179-84.

2. Bobbert P, Eisenreich A, Weithauser A, Schultheiss HP, Rauch U. Leptin and resistin induce increased procoagulability in diabetes mellitus. Cytokine. 2011;56:332-7.

3. Antoniak S, Boltzen U, Eisenreich A, Stellbaum C, Poller W, Schultheiss HP, Rauch U. Regulation of cardiomyocyte full-length tissue factor expression and microparticle release under inflammatory conditions in vitro. Journal of thrombosis and haemostasis : JTH. 2009;7:871-8.

4. Antoniak S, Boltzen U, Riad A, Kallwellis-Opara A, Rohde M, Dorner A, Tschope C, Noutsias M, Pauschinger M, Schultheiss HP, Rauch U. Viral myocarditis and coagulopathy: increased tissue factor expression and plasma thrombogenicity. Journal of molecular and cellular cardiology. 2008;45:118-26.

5. Aras O, Shet A, Bach RR, Hysjulien JL, Slungaard A, Hebbel RP, Escolar G, Jilma B, Key NS. Induction of microparticle- and cell-associated intravascular tissue factor in human endotoxemia. Blood. 2004;103:4545-53.

6. Bogdanov VY, Cimmino G, Tardos JG, Tunstead JR, Badimon JJ. Assessment of plasma tissue factor activity in patients presenting with coronary artery disease: limitations of a commercial assay. Journal of thrombosis and haemostasis : JTH. 2009;7:894-7.

7. Szotowski B, Goldin-Lang P, Antoniak S, et al. Alterations in myocardial tissue factor expression and cellular localization in dilated cardiomyopathy. Journal of the American College of Cardiology. 2005;45:1081-9.

8. Weithauser A, Bobbert P, Antoniak S, et al. Protease-activated receptor-2 regulates the innate immune response to viral infection in a coxsackievirus B3-induced myocarditis. Journal of the American College of Cardiology. 2013;62:1737-45.
