# Supplementary material for: Micro–RNA-126 Reduces the Blood Thrombogenicity in Diabetes Mellitus via Targeting of Tissue Factor
Source: Arterioscler Thromb Vasc Biol. 2016 May 25;36(6):1263–71. doi: 10.1161/ATVBAHA.115.306094 (PMC4894779; doi:10.1161/ATVBAHA.115.306094)
Supplement: Supplementary file 2 [file atv-36-1263-s002.pdf]

Supplementary Figures:

Suppl. Figure I: Gating strategy for assessment of microvesicles Megabeads (Biocytex, France) (A) were used according to the manufacturer´s recommendation to set up a size gate of < 1µm. (B) AnnexinV-binding particles (C) smaller than 1µm were considered „microvesicles“.

Suppl. Fig. II: Measurement of microvesicles derived from HMEC cells The endothelial cell markers CD144 (3rd panel in A-C) and vascular endothelial growth factor receptor 2 (4th panel A-C) were used to define the endothelial origin of the microvesicles. Fresh medium (A) was used as a control. Microvesicles in untreated (B) and TNFα-treated (C) cells were measured.

Suppl. Fig. III: miR-126 is enriched in microvesicles released by HMEC cells (A) HMEC-1 cells were cultured for 24h and then treated with TNFα for another 6h or 24h. The amount of microvesicles was subsequently measured in the cell supernatant and in the pellet fraction. (B) The endothelial cells were transfected with a control miR, miR-126 or anti-miR-126 and stimulated with TNFα for 6h. miR-126 was then quantified in the microvesicles of the transfected cells.

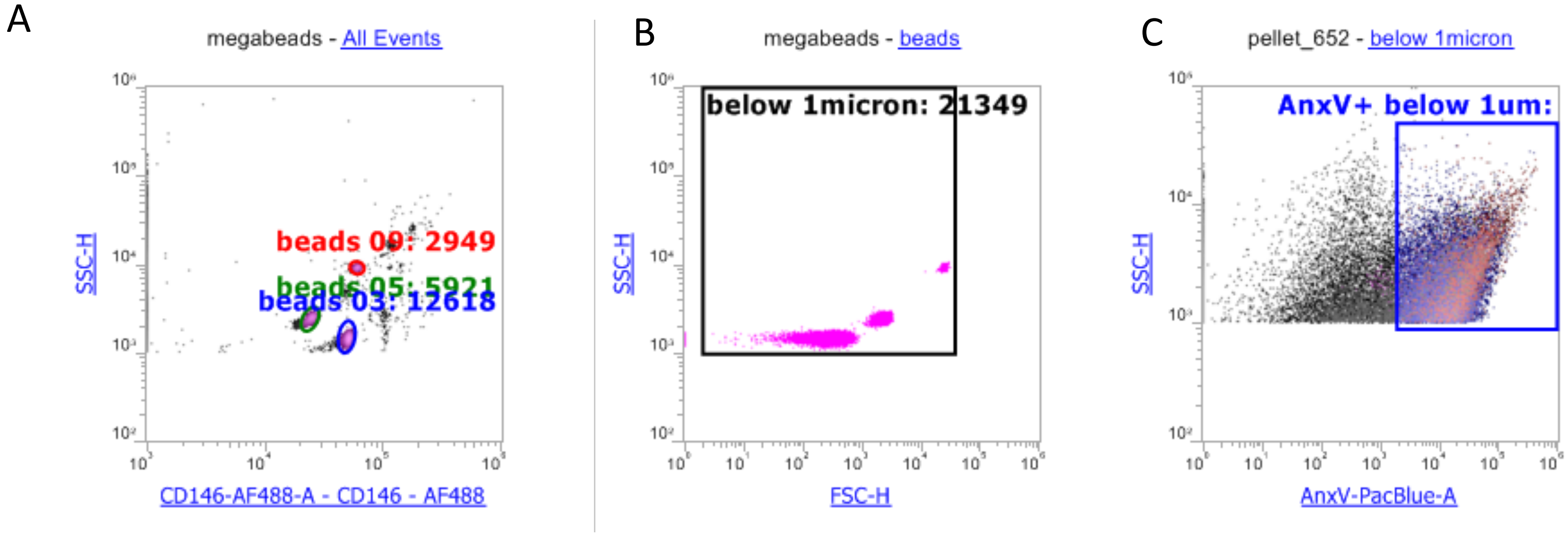
A
megabeads - All Events
beads 09: 2949
beads 05: 5921
beads 03: 12618
SSC-H
CD146-AF488-A - CD146 - AF488
B
megabeads - beads
below 1micron: 21349
SSC-H
FSC-H
C
pellet_652 - below 1micron
AnxV+ below 1um:
SSC-H
AnxV-PacBlue-A

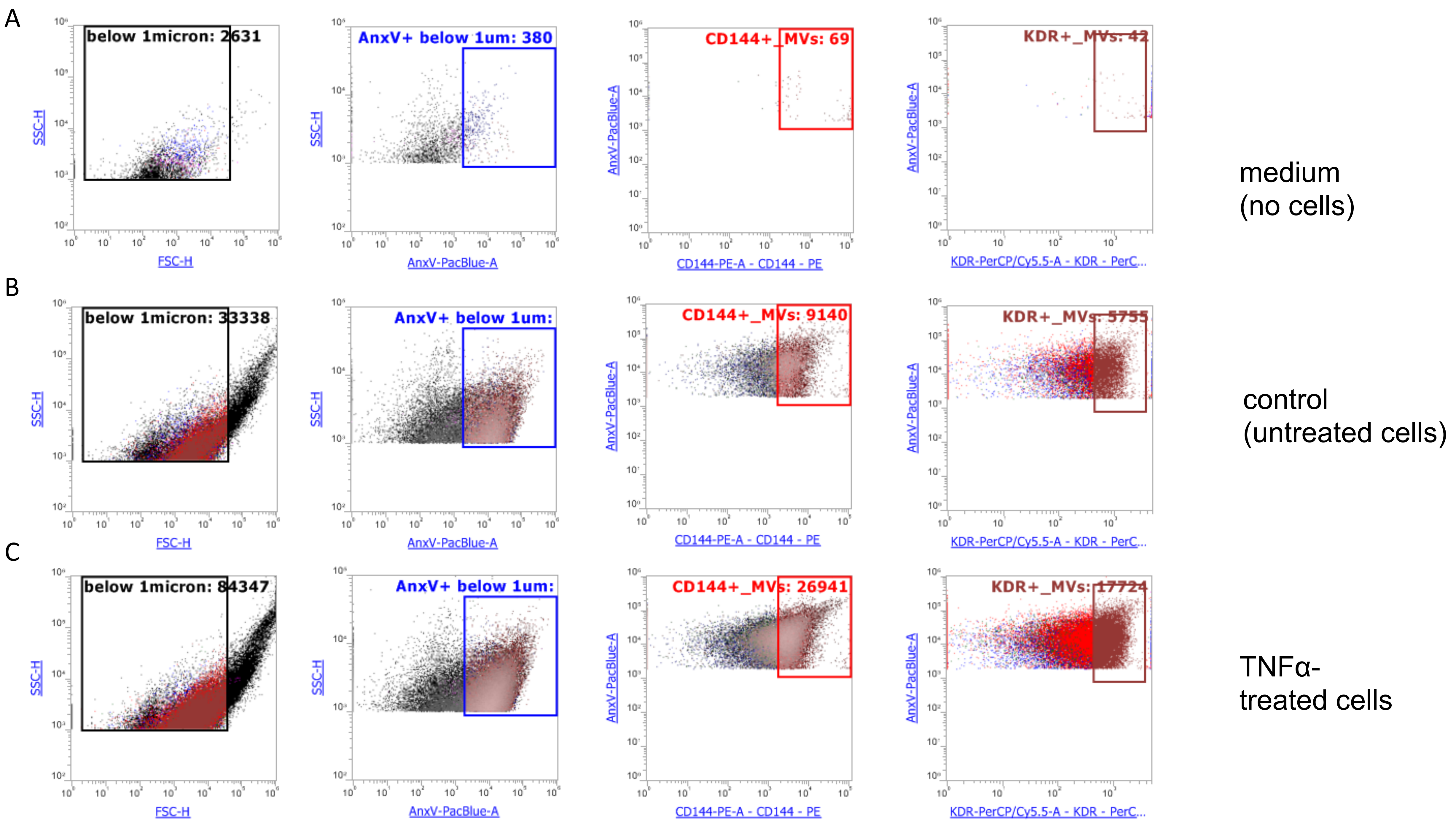
A
below 1micron: 2631
AnxV+ below 1um: 380
CD144+_MVs: 69
KDR+_MVs: 42
SSC-H
FSC-H
AnxV-PacBlue-A
CD144-PE-A - CD144 - PE
KDR-PerCP/Cy5.5-A - KDR - PerC...
medium
(no cells)
B
below 1micron: 33338
AnxV+ below 1um:
CD144+_MVs: 9140
KDR+_MVs: 5755
control
(untreated cells)
C
below 1micron: 84347
AnxV+ below 1um:
CD144+_MVs: 26941
KDR+_MVs: 17724
TNFα-
treated cells

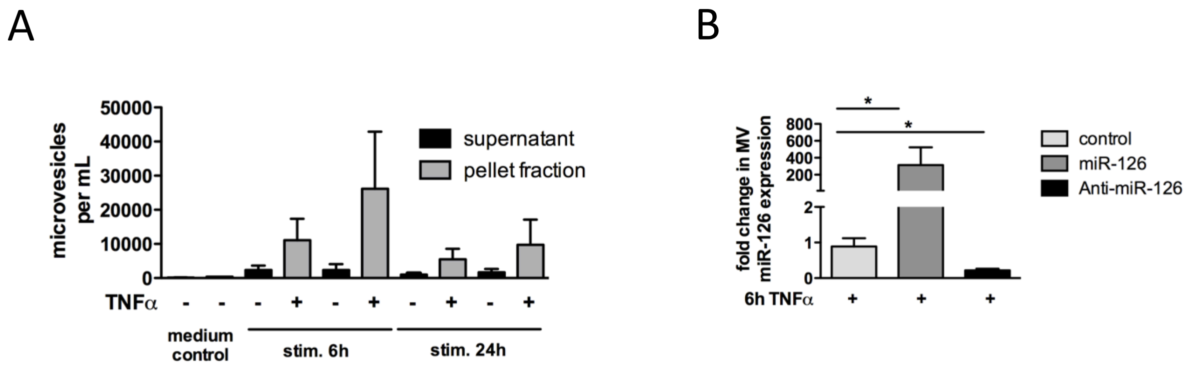

A
microvesicles per mL
50000
40000
30000
20000
10000
0
supernatant
pellet fraction
TNFα
medium control
stim. 6h
stim. 24h
B
fold change in MV miR-126 expression
800
600
400
200
2
1
0
control
miR-126
Anti-miR-126
6h TNFα
