# Supplementary figures and images for: Micro–RNA-126 Reduces the Blood Thrombogenicity in Diabetes Mellitus via Targeting of Tissue Factor
Source: Arterioscler Thromb Vasc Biol. 2016 May 25;36(6):1263–71. doi: 10.1161/ATVBAHA.115.306094 (PMC4894779; doi:10.1161/ATVBAHA.115.306094)

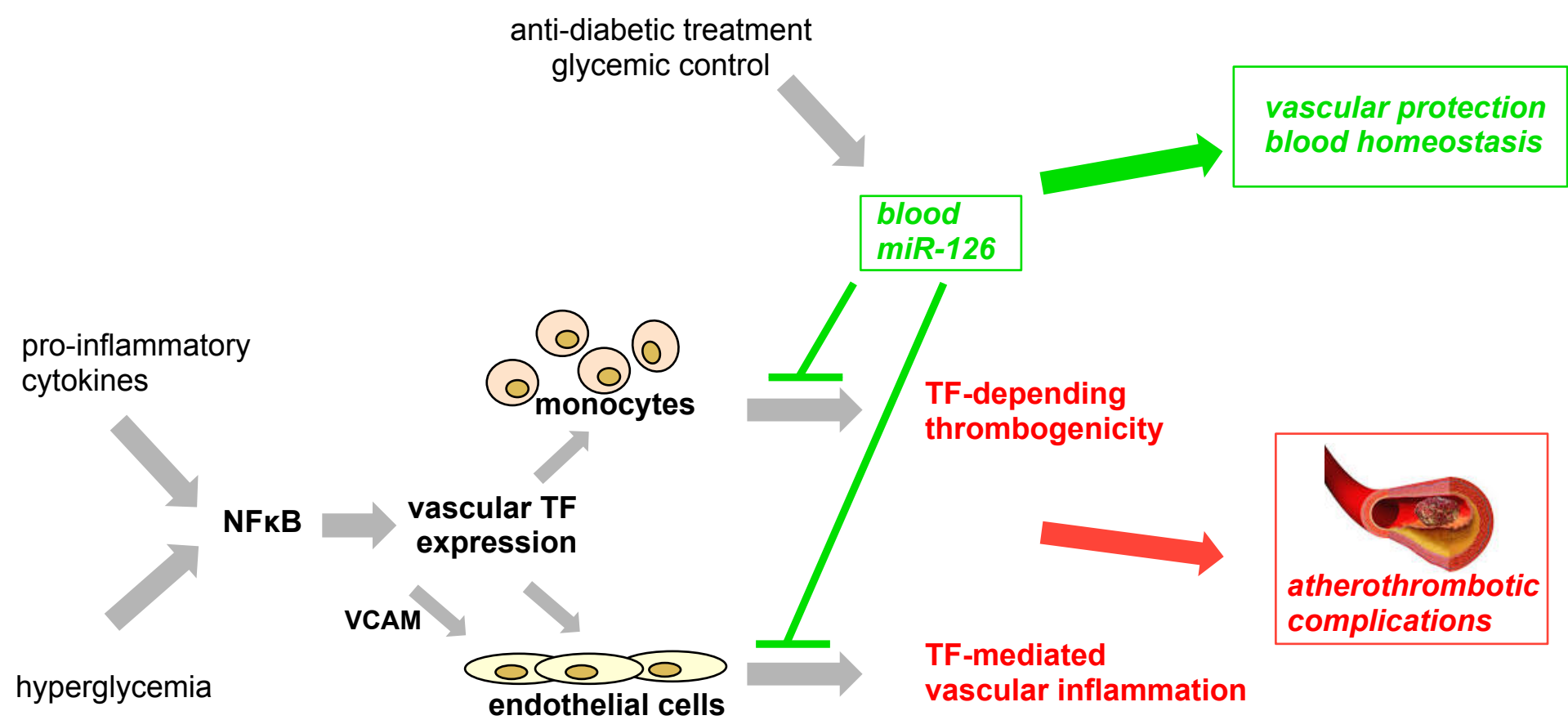

Supplement: Supplementary file 3 [file atv-36-1263-s003.pdf]
